# Integrative Network Pharmacology and Molecular Docking Analysis Uncovers Multi-Target Mechanisms of Alpha-Mangostin Against Acute Kidney Injury

**DOI:** 10.3390/foods15071270

**Published:** 2026-04-07

**Authors:** Moragot Chatatikun, Aman Tedasen, Chutima Jansakun, Passakorn Poolbua, Jason C. Huang, Jongkonnee Thanasai, Wiyada Kwanhian Klangbud, Atthaphong Phongphithakchai

**Affiliations:** 1 Department of Medical Technology, School of Allied Health Sciences, Walailak University, Nakhon Si Thammarat 80160, Thailand; moragot.ch@wu.ac.th (M.C.); aman.te@wu.ac.th (A.T.); chutima.js@wu.ac.th (C.J.); patsakorn@mail.wu.ac.th (P.P.); 2 Research Excellence Center for Innovation and Health Products (RECIHP), Walailak University, Nakhon Si Thammarat 80160, Thailand; 3 Department of Biotechnology and Laboratory Science in Medicine, National Yang Ming Chiao Tung University, Taipei 112304, Taiwan; jasonhuang@nycu.edu.tw; 4 Faculty of Medicine, Mahasarakham University, Mahasarakham 44000, Thailand; jongkonnee@msu.ac.th; 5 Medical Technology Program, Faculty of Science, Nakhon Phanom University, Nakhon Phanom 48000, Thailand; wiyadakwanhian@gmail.com; 6 Nephrology Unit, Division of Internal Medicine, Faculty of Medicine, Prince of Songkla University, Songkhla 90110, Thailand

**Keywords:** alpha-mangostin, acute kidney injury, *PTGS2*, network pharmacology, molecular docking

## Abstract

Alpha-mangostin (AM), a xanthone from *Garcinia mangostana*, has shown promising nephroprotective properties, but its mechanisms in acute kidney injury (AKI) remain incompletely defined. In this study, we applied an integrative network pharmacology pipeline combined with molecular docking to clarify AM’s multi-target mechanisms in AKI. We identified 128 predicted AM targets and intersected them with AKI-related genes, yielding 122 shared targets. Protein–protein interaction analysis identified ten hub genes—*TNF*, *AKT1*, *IL6*, *SRC*, *CTNNB1*, *HSP90AA1*, *NFKB1*, *HIF1A*, *PPARG*, and *PTGS2*—implicating inflammatory, hypoxia, and cell-survival pathways. KEGG enrichment highlighted HIF-1 signaling, PI3K–Akt signaling, chemokine signaling, AGE–RAGE signaling, and pathways related to cellular senescence and oxidative stress, while GO terms emphasized responses to chemical/oxygen-containing compounds, kinase activity, signal transduction, and apoptosis. Molecular docking against the ten hub proteins showed favorable binding energies across multiple targets. The strongest predicted affinities were observed for PTGS2 (−11.13 kcal/mol), TNF (−9.74 kcal/mol), and AKT1 (−9.48 kcal/mol). Docking positioned AM within the COX-2 catalytic pocket, engaging key catalytic and hydrophobic residues similar to known inhibitors. MD simulation interaction analysis confirmed that AM maintained stable contacts with key human PTGS2 residues, characterized by dominant hydrogen bonds and water-bridge interactions with SER353, TYR355, ARG513, and SER530, along with consistent hydrophobic contacts, and persistent interactions sustained throughout the 200 ns trajectory. Collectively, these results suggest that AM modulates interconnected inflammatory, hypoxic, and survival pathways relevant to AKI, acting as a multi-target ligand with notable interaction involving COX-2, TNF, and AKT1. Further experimental validation and formulation strategies to improve bioavailability are recommended for the advancement of AM toward therapeutic evaluation in AKI.

## 1. Introduction

Acute kidney injury (AKI) is a serious medical condition that rapidly impairs the kidneys’ ability to filter blood, maintain electrolyte balance, and support overall physiological stability. It occurs frequently among hospitalized and critically ill patients and is associated with significant complications, including higher mortality rates and long-term kidney dysfunction [1,2]. Although AKI can be triggered by various insults such as ischemia–reperfusion, severe infection, or exposure to nephrotoxic drugs, many studies show that these different causes activate similar biological processes. These processes typically involve heightened inflammation, oxidative stress, mitochondrial dysfunction, and disrupted cell-cycle regulation or apoptosis within renal tubular cells [1,2,3,4]. Recent advances in transcriptomics and single-cell analyses have revealed that stressed kidney cells adopt predictable injury states characterized by oxidative stress signaling, hypoxic responses, interferon activity, and early fibrotic changes. These recurring cellular patterns across AKI types point to shared mechanisms that could be targeted therapeutically [5,6].

In the search for new therapeutic strategies, naturally derived bioactive compounds, especially dietary phytochemicals and nutraceuticals, have attracted increasing interest because of their anti-inflammatory, antioxidant, and cytoprotective activities. One promising compound is alpha-mangostin (AM), a dietary xanthone-type phytochemical obtained from the fruit pericarp of *Garcinia mangostana*. AM has demonstrated potent immunomodulatory and anti-inflammatory activity in in vivo and in vitro models, including suppression of nuclear factor-kappa B (NF-κB) and mitogen-activated protein kinase (MAPK) signaling and attenuation of tumor necrosis factor-alpha (TNF-α) and interleukin-6 (IL-6) [7,8,9]. Emerging evidence supports these anti-inflammatory and antioxidant properties of AM in AKI. A dedicated systematic review and meta-analysis of preclinical animal studies across multiple injury models reported consistent nephroprotection by AM [10]. In those studies, AM lowered serum creatinine and blood urea nitrogen (BUN), attenuated oxidative stress markers, and reduced pro-inflammatory cytokines, with corresponding improvements in renal histopathology [9,10,11]. AM also improves kidney damage in rhabdomyolysis-induced AKI and modulates phosphoinositide 3-kinase (*PI3K*), protein kinase B (*AKT*), and c-Jun N-terminal kinase (*JNK*) in cisplatin-induced renal injury [9,12]. Together, these findings indicate that AM, which is already consumed through dietary or nutraceutical sources, may have therapeutic potential for preventing or reducing AKI-related injury. Despite accumulating evidence of AM’s nephroprotective effects in diverse AKI models, the precise molecular mechanisms underlying its therapeutic activity remain incompletely defined.

In particular, there is a lack of integrative computational analyses that connect AM’s predicted protein targets with shared injury pathways in AKI, limiting mechanistic clarity and translational potential. Network pharmacology integrates chemical information, predicted drug targets, and disease-associated genes into a single interaction network, allowing researchers to visualize compound–disease relationships, identify key regulatory hubs, and uncover potential mechanisms of action within a broader biological context [13]. Molecular docking is a complementary computational tool that supports the predictions generated from network pharmacology by examining the structural feasibility of compound–protein interactions [14]. Through simulation of the binding orientation and affinity between a ligand and a target protein, molecular docking helps to determine whether a predicted interaction is physically plausible and biologically meaningful [15]. Given its demonstrated bioactivity and accessibility as a dietary nutraceutical, AM warrants comprehensive mechanistic evaluation to clarify its therapeutic potential. This study aims to systematically characterize the potential therapeutic mechanisms of AM in AKI by identifying shared molecular targets, analyzing protein–protein interaction (PPI) networks, evaluating enriched signaling pathways, and validating drug–target interactions through molecular docking. By integrating computational pharmacology with established AKI biology, this work provides a comprehensive framework to explain how AM might modulate key injury pathways and assesses its potential as a natural therapeutic agent for the prevention or treatment of AKI.

## 2. Materials and Methods

### 2.1. Evaluation of AM’s Activity

The pharmacokinetic properties and biological activity of AM was assessed using the SwissADME and pkCSM platforms. Initially, we prepared the chemical structure of AM in SMILES format for analysis. Initially, we accessed the SwissADME platform (http://www.swissadme.ch accessed on 1 May 2025) and inputted the SMILES notation to analyze various parameters, which included physicochemical properties, adherence to Lipinski’s Rule of Five, and predictions of ADMET (absorption, distribution, metabolism, excretion, and toxicity) profiles [16]. Those results were reviewed to estimate AM’s likely bioavailability, solubility, and overall drug-likeness. Subsequently, we analyzed the same SMILES input with the pkCSM platform (https://biosig.lab.uq.edu.au/pkcsm/, accessed on 1 May 2025) to further investigate the ADMET-related properties, including absorption, distribution, metabolism, excretion, and potential toxicity [17]. Both in silico tools provided significant insights into the pharmacokinetic profile and therapeutic potential of AM.

### 2.2. Screening the Targets of AM and AKI

Alpha-mangostin (AM) targets were identified using three complementary in silico prediction tools: SwissTargetPrediction (http://www.swisstargetprediction.ch/, accessed on 2 May 2025), Super-PRED (https://prediction.charite.de/subpages/target_prediction.php/, accessed on 2 May 2025), and Similarity Ensemble Approach (SEA) (https://sea.bkslab.org/ accessed on 2 May 2025) [18]. The SMILES structure of AM was submitted to each platform, allowing target prediction based on chemical similarity (SwissTargetPrediction), ligand- and structure-based inference (Super-Pred), and molecular similarity clustering (SEA). Using multiple tools minimized platform-specific bias and increased predictive confidence. All predicted protein targets were converted to standardized gene symbols using UniProt (https://www.uniprot.org, accessed on 2 May 2025) [19]. For AKI-related genes, we retrieved the complete list of AKI-associated genes from GeneCards (https://www.genecards.org, accessed on 2 May 2025), which aggregates curated gene–disease evidence across multiple biological databases. We used the full GeneCards AKI gene set to avoid subjective filtering and ensure comprehensive coverage. To avoid overreliance on a broadly defined gene set, we intersected the GeneCards AKI genes with compound targets using a Venn diagram, thereby focusing on genes relevant to both AKI and AM. In addition, we applied the algorithm in Cytoscape to prioritize highly connected nodes and reduce or exclude weakly associated genes. After merging datasets, duplicates were removed and gene names were harmonized. This multi-database and full-inclusion strategy aligns with best practices in network-based analysis, improving the specificity and robustness of subsequent PPI and enrichment results while reducing the risk of missing relevant disease-associated genes.

### 2.3. Intersection of AM and AKI-Related Genes

To identify common genes among the identified targets of AM and the AKI-related genes, we employed the Venn diagram tool available on the Bioinformatics Portal (https://bioinformatics.psb.ugent.be/webtools/Venn/, accessed on 3 May 2025) [20]. Only genes present in both AM predictions (any of the three tools) and AKI lists were carried forward. The intersections of these gene sets were the potential targets of AM and AKI.

### 2.4. Predicting Protein–Protein Interactions (PPI) Analysis

A compiled list of genes associated with AM and AKI in *Homo sapiens* was submitted to the STRING version 12.0 database (https://string-db.org, accessed on 5 May 2025) to retrieve predicted protein–protein interactions for *Homo sapiens* using a medium-confidence score threshold (>0.4) [21]. The resulting interaction network was then exported and imported into Cytoscape 3.10.3 software (http://www.cytoscape.org accessed on 6 May 2025) for visualization and further analysis [22]. Degree centrality (DC) values were calculated to identify the top 10 hub proteins within the network, as these are indicative of the most connected proteins.

### 2.5. GO and KEGG Enrichment Analysis

To conduct Gene Ontology (GO) and Kyoto Encyclopedia of Genes and Genomes (KEGG) enrichment, analyses were performed using ShinyGO version 0.85.1 (http://bioinformatics.sdstate.edu/go/, accessed on 7 May 2025) with FDR correction (*p* < 0.05) [23]. A list of relevant genes was uploaded to ShinyGO with *Homo sapiens* selected as the organism, and both GO (biological process, molecular function, and cellular component) and KEGG pathway analyses were enabled. The results were filtered by the *p*-value cutoff to identify statistically significant terms and pathways, and enriched GO categories and KEGG pathways were retrieved for interpretation.

### 2.6. Preparation of the Three-Dimensional Structure of AM

To investigate the interaction mechanisms between AM and the candidate proteins, molecular docking was performed following the preparation of the ligand structure. The three-dimensional structure of AM was retrieved from the PubChem database (CID: 5281650, http://pubchem.ncbi.nlm.nih.gov/, accessed on 9 May 2025) in SDF format and subsequently converted to PDB format. Structural optimization was carried out using UCSF Chimera (1.17.1) by assigning bond orders, angles, and topology, adding missing and polar hydrogens at physiological pH (7.4), and performing energy minimization with 5000 steepest-descent steps (step size 0.02 Å) followed by 10 conjugate-gradient steps. The AM1-BCC algorithm from the AMBER force field was applied for charge calculation and ionization correction. Finally, missing hydrogen atoms were added using the AutoDockTools (ADT) version 1.5.7, and the optimized ligand was saved in PDBQT format for subsequent docking studies [17].

To validate the docking protocol, co-crystallized ligands from the three-dimensional structures of the target proteins were prepared and subjected to re-docking using the same computational procedures applied to the test compounds. The resulting poses were then compared with their experimentally determined crystallographic conformations to evaluate the accuracy of the docking parameters. In cases where co-crystallized ligands were not available, well-established inhibitors or reference ligands reported in the literature were selected as substitutes to ensure reliable benchmarking (Table 1). This combined approach allowed us to confirm that the docking workflow could accurately reproduce native binding orientations and provided confidence in the robustness of the protocol for subsequent ligand evaluation.

### 2.7. Preparation of Three-Dimensional Protein Structures

The target protein structures listed in Table 1 were retrieved from the RCSB Protein Data Bank (www.rcsb.org/ accessed on 30 May 2025), with preference given to crystal structures of high quality and resolutions near or below 3.0 Å. Proteins were prepared for docking by removing water molecules and co-crystallized ligands using the BIOVIA Discovery Studio. Structures were then refined by adding missing hydrogen atoms according to the protonation state at physiological pH (7.0), followed by charge assignment and atom type specification. These steps were carried out using AutoDockTools (ADT) version 4.2, ensuring accurate representation of electrostatic properties. The final protein structures were saved in PDBQT format, ready for docking simulations [18].

### 2.8. Molecular Docking Analysis Between AM and Hub Targets

Molecular docking studies were conducted using AutoDock version 4.2, employing the Lamarckian genetic algorithm to predict the binding interactions between AM and selected hub target proteins. Protein structures were treated as rigid, while the ligand was considered flexible, and default parameters in AutoDockTools (ADT) were applied for all settings. Grid parameter files (.gpf) were generated for each target protein to define the docking box dimensions, ensuring complete coverage of the receptor binding site, and processed using AutoGrid 4.2 as shown in Table 1. Docking parameter files (.dpf) were then prepared, and simulations were executed with 50 independent genetic algorithm runs, each with a population size of 200, repeated five times to enhance robustness. The optimal ligand conformations were identified based on the lowest binding energy values (kcal/mol), and binding affinity ratios were assessed by comparing AM with reference ligands. To validate the docking protocol, co-crystallized ligands from the reference protein structures (Table 1) were re-docked into their binding sites, and the predicted poses were compared with the crystallographic conformations. Low RMSD values (<3.0 Å) confirmed that the docking parameters reliably reproduced native binding orientations. In addition, the docking score of AM was compared with that of the positive control (co-crystallized ligand). The resulting protein–ligand complexes were further analyzed for binding modes and interaction profiles, and visualized using BIOVIA Discovery Studio Visualizer (Accelrys, San Diego, CA, USA) [24].

### 2.9. Molecular Dynamics Simulation

Molecular dynamics (MD) simulations were performed to investigate the time-dependent behavior of the PTGS2–AM complexes, providing insights into ligand-induced effects on protein flexibility and binding. Complex preparation at pH 7.0 was conducted using the Protein Preparation Wizard, which added hydrogens, assigned bond orders, rebuilt missing side chains and loops, optimized hydrogen-bond networks, and sampled water orientations. The OPLS4 force field was then applied. Each system was solvated in a 10 Å × 10 Å × 10 Å orthorhombic TIP3P water box, neutralized with 0.15 M Na^+^ and Cl^−^ ions, and parameterized for simulation. Production runs were carried out for 200 ns under an NPT ensemble at 310 K and 1.01 bar, with long-range electrostatics calculated using the Smooth Particle Mesh Ewald (PME) method and solvent represented by a simple point-charge model. Trajectory analyses were performed using the Simulation Interaction Diagram wizard, including RMSD profiles, RMSF plots, ligand–protein contact maps, and timeline interaction analyses. All simulations and analyses were conducted using Desmond (Schrödinger), providing a comprehensive assessment of structural stability, conformational dynamics, and key interaction hotspots throughout the simulation.

## 3. Results

### 3.1. AM’s Activity

AM demonstrated properties characteristic of a highly lipophilic, poorly soluble small molecule as shown in Supplementary Table S1. AM (C_24_H_26_O_6_) has a molecular weight of 410.46 g/mol. SwissADME predicted 30 heavy atoms, 14 aromatic atoms, a low fraction Csp^3^ (0.29), 6 hydrogen-bond acceptors (HBAs), 3 hydrogen-bond donors (HBDs), and a topological polar surface area (TPSA) of 100.13 Å^2^. Lipophilicity was high (consensus Log P = 4.64), and all solubility models (ESOL, Ali, and SILICOS-IT) classified the compound as poorly soluble. Pharmacokinetic predictions indicated high gastrointestinal (GI) absorption but low Caco-2 permeability and P-glycoprotein (P-gp) substrate behavior. Distribution parameters suggested strong plasma protein binding (an extremely low fraction unbound) and poor blood–brain barrier (BBB) and central nervous system (CNS) permeability (logBB = −1.075; logPS = −1.984). Metabolism modeling identified AM as a cytochrome P450 3A4 (CYP3A4) substrate and a predicted inhibitor of CYP1A2, CYP2C19, and CYP2C9. pkCSM estimated moderate clearance (0.43 mL/min/kg) and no interaction with renal organic cation transporter 2 (OCT2). Toxicity modeling showed AMES mutagenicity and hERG II inhibition but no hepatotoxicity or skin sensitization. Drug-likeness filters (Lipinski, Ghose, Veber, and Egan) were satisfied except for the Muegge criteria due to high lipophilicity, and the compound showed no pan-assay interference substances alerts (PAINS), though two Brenk alerts were noted. Overall, these in silico results portray AM as a lipophilic, poorly soluble compound with good predicted oral absorption but limited permeability and brain access, strong protein binding, CYP-mediated interaction potential, some toxicity concerns, and generally acceptable drug-like properties.

### 3.2. Target Genes of AM and AKI

A total of 128 predicted targets of AM were identified using SwissTargetPrediction, Super-PRED, and Similarity Ensemble Approach (SEA). These targets were converted to gene names through UniProt. GeneCards yielded 11,630 target genes associated with AKI.

### 3.3. Common Target Genes of AM and AKI

**Figure 1:**
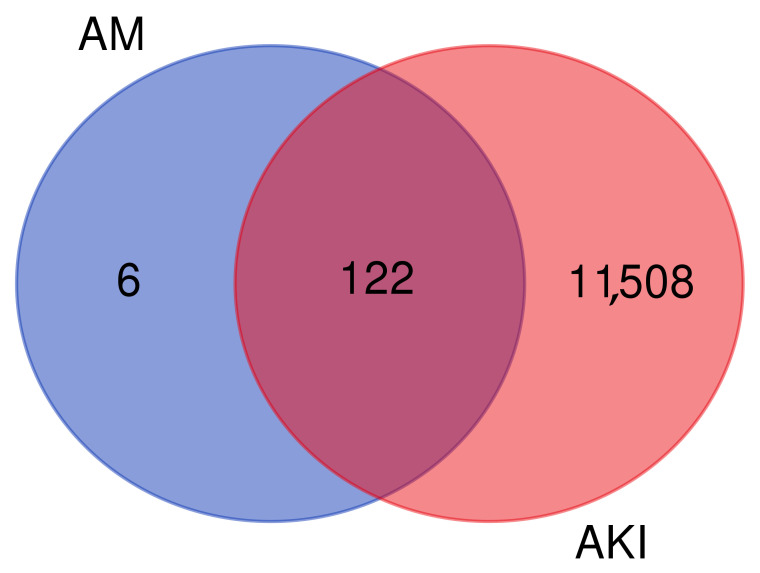
Venn diagram showing overlap between predicted targets of alpha-mangostin (AM) and acute kidney injury (AKI)-related genes.

A total of 128 targets of AM were identified, of which 122 overlap with genes associated with AKI, leaving 6 targets unique to AM. GeneCards returned 11,630 AKI-related genes in total, of which 11,508 were not shared with the predicted AM targets. Therefore, 122 genes represent the intersection between AM targets and AKI-related genes as shown in Figure 1 and Table 2.

### 3.4. Construction of AM-AKI Network

**Figure 2:**
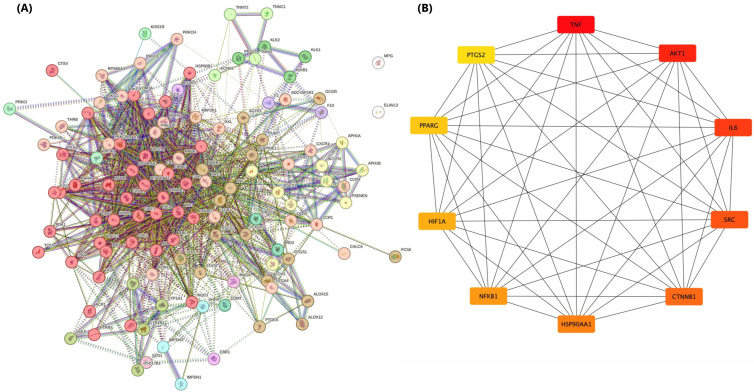
(**A**) Protein–protein interaction (PPI) network of 122 overlapping targets of alpha-mangostin (AM) and acute kidney injury (AKI). PPI network constructed from proteins shared between AM targets and AKI-associated proteins. Nodes represent proteins and edges represent reported or predicted interactions (STRING database). (**B**) Subnetwork of the top 10 hub genes ranked by degree centrality (DC) values: *TNF*, *AKT1*, *IL6*, *SRC*, *CTNNB1*, *HSP90AA1*, *NFKB1*, *HIF1A*, *PPARG* and *PTGS2*. Node color intensity corresponds to centrality (red to yellow), and edge thickness reflects interaction strength.

The 122 overlapping targets between AM and AKI were considered as potential therapeutic nodes and submitted to the STRING database to construct a PPI network for *Homo sapiens* (Figure 2A). The PPI network was generated using an interaction score threshold of 0.400 (medium confidence) and comprised 120 nodes and 1236 edges, with an average node degree of 20.6, indicating densely connected proteins. The network’s average local clustering coefficient was 0.597, reflecting a high tendency for connected proteins to form tight clusters. Compared with the expected 559 edges, the observed 1236 edges represented a substantial excess of interactions: the PPI enrichment *p*-value was <1.0 × 10^−16^. These metrics indicate that the AM-AKI-associated proteins are significantly more interconnected than expected by chance, suggesting coherent functional relationships relevant to disease mechanisms.

CytoHubba identified the top 10 hub genes in the AM–AKI network: tumor necrosis factor (*TNF*), AKT serine/threonine kinase 1 (*AKT1*), interleukin 6 (*IL6*), SRC proto-oncogene, non-receptor tyrosine kinase (*SRC*), catenin beta 1 (*CTNNB1*), heat shock protein 90 alpha family class A member 1 (*HSP90AA1*), nuclear factor kappa B subunit 1 (*NFKB1*), hypoxia-inducible factor 1 subunit alpha (*HIF1A*), peroxisome proliferator-activated receptor gamma (*PPARG*), and prostaglandin-endoperoxide synthase 2 (*PTGS2*, also known as cyclooxygenase-2 (COX-2)) as shown in Figure 2B. These genes represent the most highly connected nodes in the network and likely play central roles in the molecular interactions underlying AM and AKI. In the network visualization, node color ranges from red to yell in a gradient with deeper red indicating higher CytoHubba scores and lighter yellow indicating lower scores among the top ten. These hub targets may play significant roles in the mechanisms by which AM influences AKI.

### 3.5. KEGG and GO Enrichment Analysis

#### 3.5.1. KEGG Pathway Enrichment Analysis

**Figure 3:**
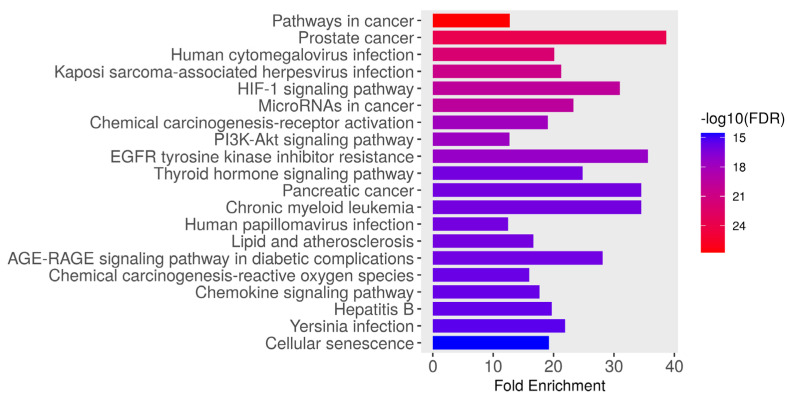
Kyoto Encyclopedia of Genes and Genomes (KEGG) pathway enrichment analysis of genes associated with alpha-mangostin (AM) and acute kidney injury (AKI). The bar plot displays the top enriched pathways ranked by fold enrichment (x-axis), with bar color indicating pathway significance as –log10(FDR) (color scale on right; redder colors = more significant).

A set of 122 overlapping genes were subjected to KEGG analysis, which identified the top 20 pathways for these target genes based on their fold enrichment and −log10(FDR) values as shown in Figure 3 and Table 3. The −log10(FDR) estimates the statistical significance of each pathway; a higher −log10(FDR) indicates greater significance. The top 20 pathways include pathways in cancer (hsa05200), prostate cancer (hsa05215), human cytomegalovirus infection (hsa05163), Kaposi sarcoma-associated herpesvirus infection (hsa05167), and the HIF-1 signaling pathway (hsa04066). Other strongly enriched pathways include microRNAs in cancer (hsa05206), chemical carcinogenesis—receptor activation (hsa05207), PI3K–Akt signaling (hsa04151), EGFR tyrosine kinase inhibitor resistance (hsa01521), and thyroid hormone signaling (hsa04919). Additional notable enrichments included pancreatic cancer and chronic myeloid leukemia (hsa05212), human papillomavirus infection (hsa05165), lipid and atherosclerosis (hsa05417), AGE–RAGE signaling in diabetic complications (hsa04933), chemical carcinogenesis—reactive oxygen species (hsa05208), chemokine signaling (hsa04062), hepatitis B (hsa05161), Yersinia infection (hsa05135), and cellular senescence (hsa04218). Overall, these results indicate that AM–AKI shared targets are highly enriched in oncogenic signaling, hypoxia and survival pathways (HIF-1, PI3K–Akt, and EGFR), inflammatory and chemokine signaling, cellular senescence, and stress/oxidative response processes (AGE–RAGE, chemical carcinogenesis), suggesting mechanistic links between AM activity and pathways relevant to kidney injury and cellular stress responses.

#### 3.5.2. GO Biological Process Enrichment Analysis

**Figure 4:**
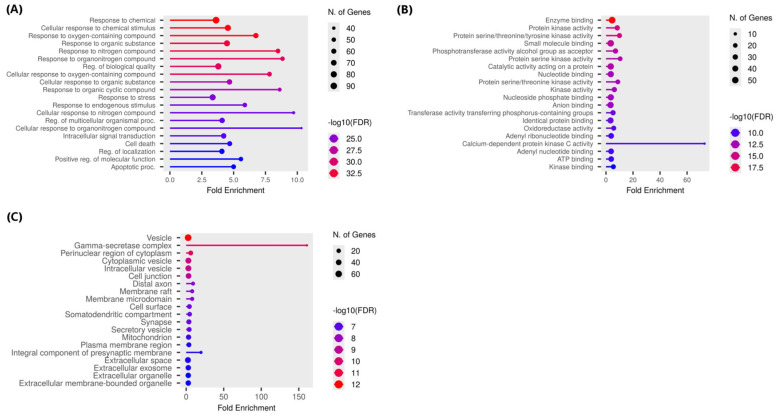
Gene Ontology (GO) enrichment analysis including (**A**) biological process, (**B**) molecular function, and (**C**) cellular components. Dot plot of enriched GO terms showing fold enrichment (x-axis) for each term (y-axis). Point size corresponds to the number of genes annotated to the term, and color indicates significance (−log10 FDR; hotter colors = more significant).

The GO enrichment results of the target gene set are presented in Figure 4A and Table 4. The most significant GO terms included response to chemical (GO:0042221; FDR 3.9104 × 10^−35^, fold enrichment 3.62), cellular response to chemical stimulus (GO:0070887; FDR 1.0511 × 10^−34^, fold enrichment 4.55), response to oxygen-containing compound (GO:1901700; FDR 1.2268 × 10^−34^, fold enrichment 6.74), response to organic substance (GO:0010033; FDR 3.3062 × 10^−33^, fold enrichment 4.48), and response to nitrogen compound/organonitrogen compound (GO:1901698, GO:0010243; FDRs 5.8736 × 10^−33^ and 1.2324 × 10^−31^, fold enrichments 8.48 and 8.84). Additional enriched categories included regulation of biological quality (GO:0065008), cellular response to oxygen-containing compound (GO:0071310), cellular response to organic substance (GO:0071310), response to organic cyclic compound (GO:0014070), response to stress (GO:0006950), response to endogenous stimulus (GO:0009719), response to endogenous stimulus (GO:0009719), cellular response to nitrogen compound (GO:1901699), regulation of multicellular organismal process (GO:0051239), cellular response to organonitrogen compound (GO:0071417), intracellular signal transduction (GO:0035556), cell death (GO:0008219), regulation of localization (GO:0032879), positive regulation of molecular function (GO:0044093), and apoptotic process (GO:0006915) (all FDRs ≤ 2.32 × 10^−23^). These results suggest the gene set is strongly associated with cellular responses to chemical and oxidative/nitrogenous stress and with intracellular signaling networks that regulate survival, inflammation and programmed cell death.

#### 3.5.3. GO Molecular Function Enrichment Analysis

GO molecular function enrichment analysis of the target gene set revealed strong and highly significant enrichment for binding and catalytic activities central to signaling and metabolic regulation as shown in Figure 4B and Table 5. The most significant term was enzyme binding (GO:0019899; FDR = 2.3224 × 10^−20^, fold enrichment = 4.53). Kinase-related activities were highly enriched, including protein kinase activity (GO:0004672; FDR = 8.1332 × 10^−16^, fold enrichment = 8.31), protein serine/threonine/tyrosine kinase activity (GO:0004712; FDR = 8.1332 × 10^−16^, fold enrichment = 9.98), protein serine kinase activity (GO:0106310; FDR = 9.5501 × 10^−14^, fold enrichment = 10.47), protein serine/threonine kinase activity (GO:0004674; FDR = 4.32 × 10^−13^, fold enrichment = 8.76), and kinase activity/binding terms (GO:0016301, GO:0019900; FDRs ≤ 4.3208 × 10^−13^, folds 6.19 and 5.46). Small molecule and nucleotide-binding functions were prominent (small molecule binding GO:0036094; nucleotide/adenyl/ATP binding GO:0000166, GO:0032559, GO:0030554, GO:0005524). Transferase and phosphotransferase activities were enriched (GO:0016773, GO:0016772), as were oxidoreductase activities (GO:0016491) and anion binding (GO:0043168). A notable highly specific hit was calcium-dependent protein kinase C activity (GO:0004698) with very high fold enrichment (72.94). Overall, these results indicate the gene set is strongly biased toward kinase-driven signal transduction, nucleotide/ATP-dependent processes, transferase and oxidoreductase activities, and diverse binding functions relevant to cellular signaling, metabolism, and stress responses.

#### 3.5.4. GO Cellular Component Enrichment Analysis

GO cellular component enrichment ranked by FDR identified vesicle-associated terms (GO:0031982) as the most significant (vesicle, FDR = 9.3242 × 10^−13^, fold enrichment = 2.65), with the gamma-secretase complex showing the largest fold enrichment (GO:0070765; FDR = 3.2603 × 10^−11^, fold enrichment = 160.76), followed by perinuclear region of cytoplasm (GO:0048471), cytoplasmic vesicle (GO:0031410), intracellular vesicle (GO:0097708), cell junction (GO:0030054), distal axon (GO:0150034), membrane raft (GO:0045121), membrane microdomain (GO:0098857), cell surface (GO:0009986), somatodendritic compartment (GO:0036477), synapse (GO:0045202), secretory vesicle (GO:0099503), mitochondrion (GO:0005739), plasma membrane region (GO:0098590), integral component of presynaptic membrane (GO:0099056), extracellular space (GO:0005615), extracellular exosome (GO:0070062), extracellular organelle (GO:0043230), and extracellular membrane-bounded organelle (GO:0065010) as shown in Figure 4C and Table 6. These results indicate that AM–AKI intersecting targets are enriched in vesicle- and membrane-associated compartments, with a notable, highly specific signal for the gamma-secretase complex.

### 3.6. Molecular Docking Results

Molecular docking was performed against ten hub proteins identified as key therapeutic targets including *AKT1*, *IL6*, *SRC*, *CTNNB1*, *HSP90AA1*, *NFKB1*, *HIF1A*, *PPARG*, *PTGS2*, and *TNF*. As shown in Table 7, the re-docking of co-crystallized ligands yielded low RMSD values (<3.0 Å), confirming that the docking parameters reliably reproduced the native binding orientations of the positive controls. In addition, the docking score of AM was compared with that of the co-crystallized ligand, further supporting the validity of the docking protocol. The molecular docking analysis revealed that AM exhibited favorable binding affinities across these targets, with binding energies ranging from −4.76 to −11.13 kcal/mol (Table 7). The strongest predicted interaction was observed with PTGS2 (COX-2), where AM exhibited a binding energy of −11.13 kcal/mol and an estimated inhibition constant of 6.95 nM against human PTGS2, surpassing the affinity of the co-crystallized reference inhibitor rofecoxib (−10.55 kcal/mol, 18.63 nM) and showing stronger binding than celecoxib in the murine PTGS2 structure (−10.84 kcal/mol, 11.32 nM). Similarly, AM demonstrated strong binding to TNF (−9.74 kcal/mol, 72.51 nM) and AKT1 (−9.48 kcal/mol, 112.82 nM), though the positive controls (D84201 and IQO0444, respectively) showed comparatively higher affinities. Notable binding was observed for HSP90AA1 (−9.16 kcal/mol, 193.81 nM) and HIF1A (−8.88 kcal/mol, 309.33 nM), which might suggest potential modulation of stress-response and hypoxia signaling. Moderate interactions were found for SRC (−8.64 kcal/mol, 464.16 nM), PPARG (−8.24 kcal/mol, 909.09 nM) and NFKB1 (−7.31 kcal/mol, 4350.00 µM), while weaker binding was detected for IL6 (−6.67 kcal/mol) and CTNNB1 (−4.76 kcal/mol) relative to their positive controls. Overall, these in silico results indicate the multi-target binding potential of AM, and particularly strong interactions with PTGS2, TNF, and AKT1, supporting the role it might serve as a candidate for modulating AKI-related signaling pathways.

### 3.7. Molecular Docking Results of PTGS2 with AM and Positive Control (Rofecoxib and Celecoxib)

**Figure 5:**
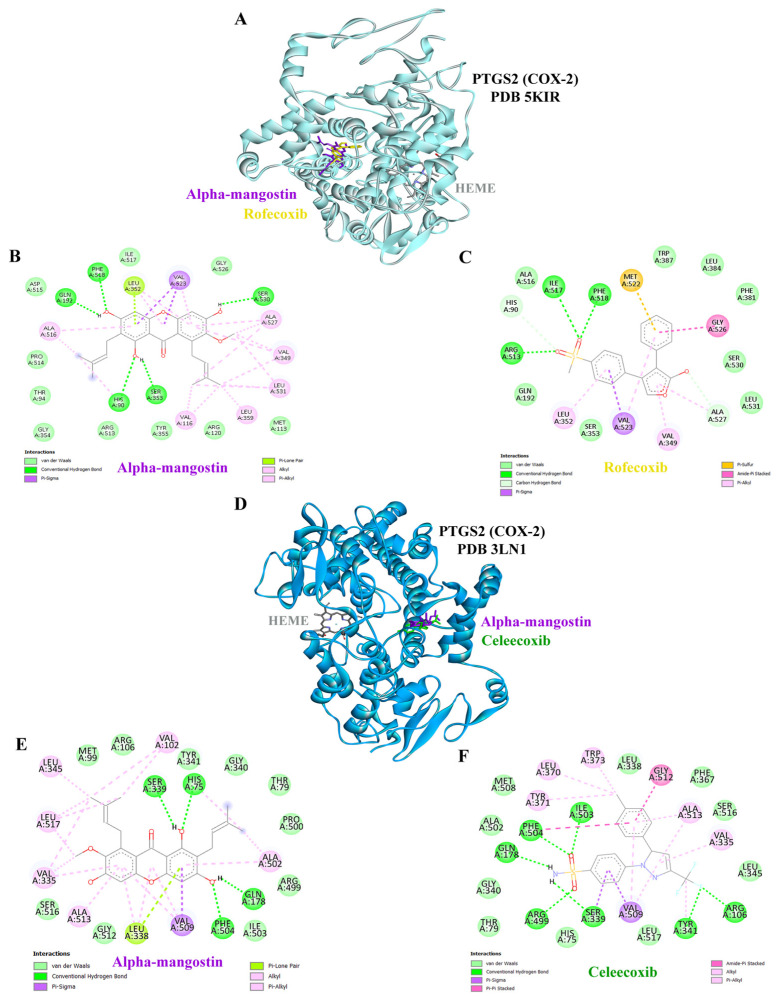
Molecular docking interactions of alpha-mangostin (AM) and rofecoxib with human PTGS2 (COX-2). (**A**) Three-dimensional structure of human COX-2 protein (PDB 5KIR) complexed with AM (purple) and rofecoxib (yellow). Detailed 2D interaction views showing key amino acid residues involved in binding with (**B**) AM and (**C**) rofecoxib. (**D**) Three-dimensional structure of murine COX-2 protein (PDB 3LN1) complexed with AM (purple) and celecoxib (green). Two-dimensional interaction maps illustrating the binding profiles of (**E**) AM and (**F**) celecoxib (positive control) with murine COX-2 active site.

Docking analysis revealed that both AM and the reference inhibitor rofecoxib occupied the active-site pocket of human PTGS2 (COX-2), while AM also bound at the same pocket site as the clinical drug celecoxib in murine PTGS2, engaging key residues essential for enzymatic activity (Figure 5A,D). In the three-dimensional structural view, AM was positioned stably within the catalytic cleft, forming multiple hydrogen bonds with HIS90, GLN192, SER353, PHE518 and SER530, which contributed to its binding stability. AM also established hydrophobic contacts with key residues such as VAL116, VAL349, LEU352, LEU359, ALA516, LEU531, VAL523 and ALA527 (Figure 5B). Rofecoxib bound in a similar region of the active site and displayed a comparable interaction profile, including hydrophobic and hydrogen bond contacts with the same core residues (Figure 5A,C). The two-dimensional interaction diagrams comparing AM with celecoxib in murine PTGS2 confirmed that AM adopts a compact binding mode, forming several strong hydrogen bonds with the same key amino acids (Figure 5E,F). Collectively, these findings demonstrate that AM might engage the PTGS2 active site in a manner analogous to rofecoxib and celecoxib, supporting its potential as a natural COX-2 inhibitor.

### 3.8. Molecular Docking Results of TNF and AKT1 with AM

**Figure 6:**
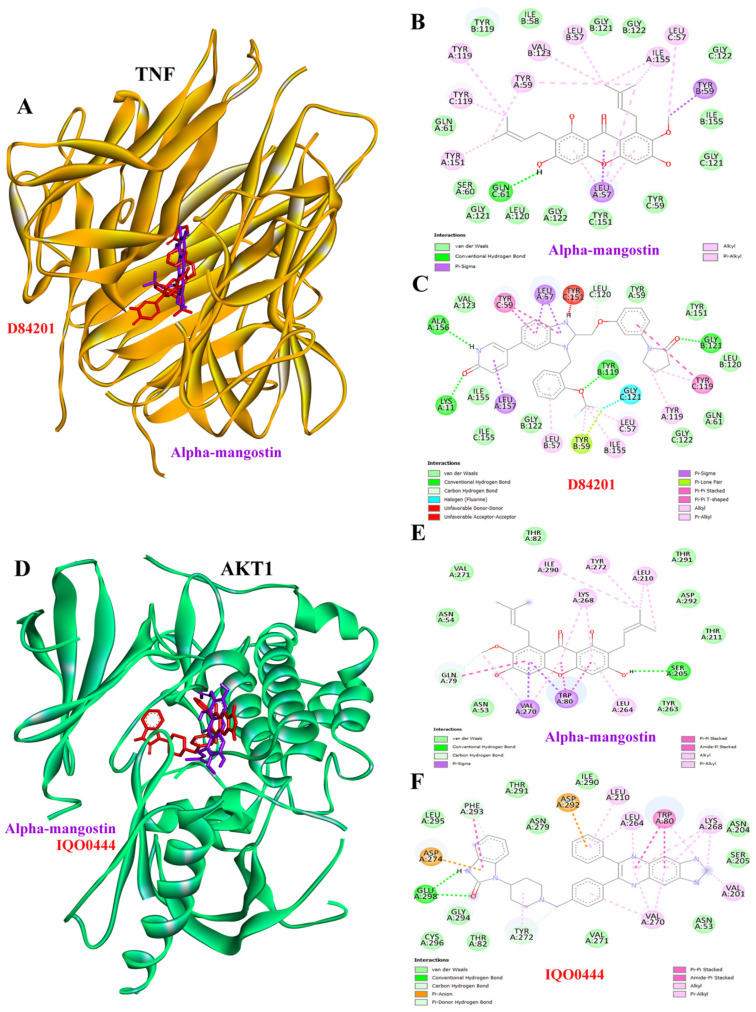
Molecular docking interactions of alpha-mangostin (AM) with TNF and AKT1. Three-dimensional structure of (**A**) TNF protein complexed with AM (purple) and D84201 (red). Two-dimensional interaction maps illustrate the binding profiles of AM (**B**) and D84201 (**C**) with TNF active site. Three-dimensional structure of (**D**) AKT1 protein complexed with AM (purple) and IQO0444. Two-dimensional interaction maps illustrate the binding profiles of AM (**E**) and IQO0444 (**F**) with the AKT1 active site.

Molecular docking analysis revealed that AM exhibited stable binding interactions with both TNF and AKT1 proteins at their respective active sites, using D84201 and IQO0444 as positive controls, respectively (Figure 6A,D). In TNF, AM occupied the active-site pocket and formed one hydrogen bond with GLN61 and several hydrophobic contacts with key residues such as LEU57, TYR59, TYR19, TYR151, VAL123, and ILE155, suggesting favorable stabilization of the complex (Figure 6B). D84201, a co-crystallized positive control, formed five hydrogen bonds with LYS11, TYR119, LEU120, GLY121, and ALA156 (Figure 6C). Similarly, AM bound within the active site of AKT1, engaging residues including GLN79 and SER205 through two hydrogen bonds, together with hydrophobic interactions involving TRP80, LEU210, LEU264, LYS268, VAL270, TYR272 and IIE290 as shown in Figure 6E. IQO0444, a co-crystallized positive control, formed four hydrogen bonds with GLU298, PHE293 and TYR272 (Figure 6F). These results indicate that AM might interact with key residues in both TNF and AKT1, supporting its potential role in modulating inflammatory and AKI signaling pathways.

### 3.9. Molecular Dynamic (MD) Simulation Analysis

**Figure 7:**
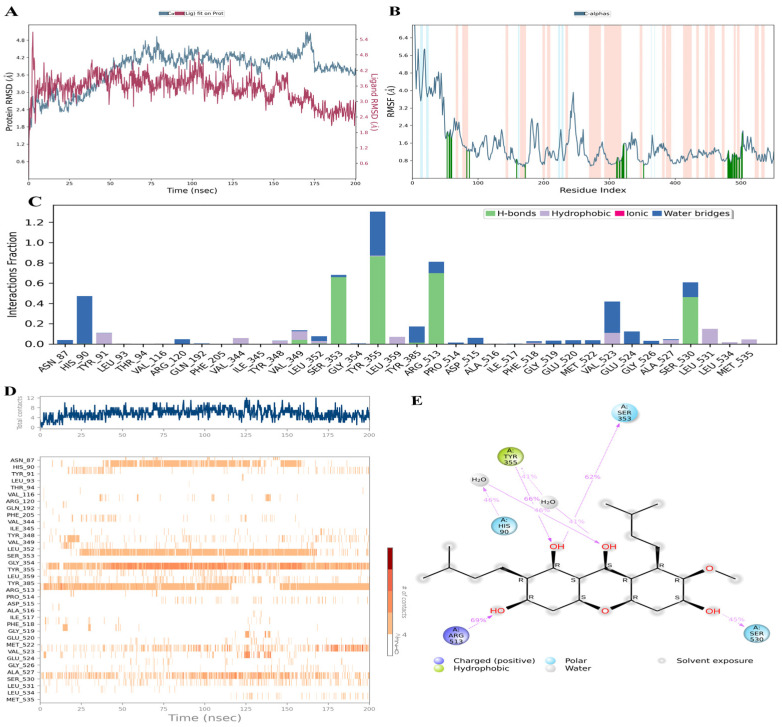
Molecular dynamics (MD) simulation of the AM–PTGS2 complex over a 200 ns trajectory. (**A**) Root mean square deviation (RMSD) of the protein backbone and ligand over the simulation period, indicating the overall structural stability of the complex. (**B**) Root mean square fluctuation (RMSF) per residue, showing the flexibility of amino acid residues during the simulation. The colored regions highlight different protein segments, where higher RMSF values (green shade regions) indicate more flexible residues and lower RMSF values (orange shade regions) indicate more stable regions. (**C**) Timeline interaction analysis across the simulation. (**D**) Post-simulation contact maps depicting amino acid interactions. (**E**) Two-dimensional schematic diagram illustrating the major interactions between alpha-mangostin (AM) with prostaglandin-endoperoxide synthase 2 (PTGS2).

AM emerged as the most promising candidate due to its strong binding affinity with key PTGS2 (COX-2) residues and its high docking score, with its interaction profile further validated through 200 ns molecular dynamics simulations. The MD trajectory revealed stable binding and favorable interaction dynamics with PTGS2. The protein RMSD stabilized within 3.6–4.8 Å during the 75–200 ns interval, indicating overall structural stability. Minor fluctuations were observed between 160 and 175 ns, but these did not exceed 3.0 Å, confirming the robustness of the complex (Figure 7A). RMSF analysis revealed moderate fluctuations across PTGS2 residues, with pronounced peaks reaching ~4 Å in the 241–250 residue range, corresponding primarily to flexible loop regions. In contrast, structured domains such as α-helices and β-sheets remained comparatively stable, reflecting localized flexibility without compromising the overall structural integrity of the protein (Figure 7B). Interaction fraction analysis revealed that AM formed stable contacts with several key PTGS2 residues. Hydrogen bonds and water-bridge interactions were dominant, particularly with SER353, TRY355, ARG513 and SER530, while hydrophobic contacts were consistently observed with VAL523 and LEU531 (Figure 7C). Time-dependent contact analysis demonstrated that AM maintained persistent interactions with several key PTGS2 residues throughout the 200 ns trajectory. Notably, residues such as SER353, TYR355, and ARG513 exhibited frequent and stable contacts, underscoring their importance in ligand recognition and binding stability (Figure 7D). The 2D interaction diagram further highlighted dominant hydrogen-bonding, hydrophobic interaction and polar contacts, with TYR355, ARG513, and SER353 and SER530 showing the highest interaction frequencies (Figure 7E). Collectively, these findings confirm that AM might engage PTGS2 through a combination of hydrogen bonds, polar interactions, and water bridges, ensuring durable and favorable binding dynamics.

## 4. Discussion

This study examined AM’s potential therapeutic effects in AKI through an integrative approach combining network pharmacology and molecular docking. Intersection analysis and PPI construction identified key inflammatory and stress-response hubs such as *TNF*, *IL-6*, *AKT1*, *PTGS2 (COX-2)*, *NFKB1*, *HIF1A*, *HSP90AA1*, *PPARG*, *SRC*, and *CTNNB1*.

AM shows promising therapeutic potential despite challenges from its high lipophilicity and poor solubility, which are common challenges in drug development. Its high lipophilicity enhances gastrointestinal absorption, though overcoming solubility issues remains critical. Such properties necessitate advanced formulation strategies, such as cyclodextrin inclusion complexes, nanoemulsions, polymeric nanoparticles, micelles, and solid dispersions to achieve therapeutic concentrations [25,26]. The compound’s strong protein binding is typical of lipophilic molecules and could limit its free concentration in plasma, which is pivotal for efficacy. Exploring methods to modulate this binding or enhance its delivery in therapeutic contexts like AKI might offer solutions [27]. Regarding safety and metabolism, AM’s interactions with cytochrome P450 enzymes (CYP450) highlight the importance of considering potential drug–drug interactions, especially in patients with complex medication regimens. This requires careful monitoring and possibly personalized dosing strategies to mitigate risks [28]. A positive AMES prediction suggests only a potential risk of genotoxicity, which must be confirmed experimentally using standardized assays (Ames, micronucleus, or Comet tests) to determine whether the compound induces DNA damage under biological conditions [29]. Similarly, predicted hERG II inhibition indicates a theoretical possibility of cardiotoxic liability, but hERG II models are less specific than hERG I and often over-predict risk; thus, electrophysiological studies such as patch-clamp assays are required to establish whether AM truly affects cardiac ion channels [30]. Predicted ADMET properties for AM indicate several potential bioavailability limitations, including high lipophilicity, poor aqueous solubility, extensive plasma-protein binding and variable permeability. These values are modeled estimates and do not account for formulation effects, first-pass metabolism, transporter activity, active metabolite formation or inter-individual and inter-species variability. Consequently, these predictions require experimental verification through solubility testing, Caco-2 or Parallel Artificial Membrane Permeability Assay (PAMPA) permeability assays, plasma-protein binding studies, microsomal or hepatocyte metabolism assays and in vivo pharmacokinetic evaluation before reliable conclusions about oral exposure [31,32,33]. Overall, these in silico alerts should be interpreted as hypothesis-generating rather than definitive indicators of human toxicity [34]. Although AM shows promising multi-target activity in AKI, its potential mutagenicity, cardiotoxicity, and poor bioavailability remain key barriers to translation, underscoring the need for optimized delivery strategies, detailed pharmacokinetic characterization, and rigorous toxicological assessment to define safe and effective dosing.

The identification of 122 overlapping genes between AM and AKI revealed its potential to influence critical pathways involved in the pathophysiology of renal injury. Using a network-pharmacology pipeline integrated with molecular docking, we identified a sizeable intersection between predicted targets of AM and AKI-associated genes and prioritized ten hubs (*TNF*, *AKT1*, *IL6*, *SRC*, *CTNNB1*, *HSP90AA1*, *NFKB1*, *HIF1A*, *PPARG*, and *PTGS2*). The biological relevance of the ten hub genes is well supported by the current AKI literature. *TNF* is a major pro-inflammatory cytokine that drives renal tissue damage and worsens experimental nephrotoxic injury, while *IL6* activates NF-κB/STAT3 signaling and amplifies cytokine-driven inflammation involved in tubular injury [3,35]. *AKT1* regulates PI3K-dependent survival and metabolic pathways activated during renal hypoxia and inflammation, and HIF1A mediates hypoxia-induced transcription and metabolic adaptation, a hallmark of ischemic AKI [36,37]. *PTGS2* (COX-2) modulates prostaglandin synthesis and contributes to inflammatory and hemodynamic injury in kidney damage [38]. *SRC*, *CTNNB1*, and *HSP90AA1* participate in inflammation, stress signaling, Wnt/β-catenin regulation and protein stabilization during hypoxic and inflammatory injury states [39,40,41]. *NFKB1* is a central driver of inflammatory gene expression and leukocyte recruitment in renal injury, while *PPARG* regulates metabolic and inflammatory responses implicated in tubular stress and renal injury [42,43]. Together, these findings confirm that the selected hub proteins are key regulators of inflammation, hypoxia, metabolic stress and cell-survival pathways central to AKI pathogenesis.

KEGG analysis returned HIF-1 signaling, PI3K–Akt, EGFR tyrosine kinase inhibitor resistance, chemokine signaling, AGE–RAGE, chemical carcinogenesis/ROS, and cellular senescence among the top terms, an expected palette given that ischemia, sterile inflammation and oxidative stress dominate canonical AKI biology. The prominence of *HIF-1* and PI3K–Akt is mechanistically coherent; HIF-1 coordinates glycolytic shift, mitophagy and survival programs during ischemia–reperfusion, while PI3K–Akt integrates survival and inflammatory signaling in injured tubular cells [44,45]. Within this broad enrichment landscape, a more focused interpretation highlights HIF-1 signaling and PI3K–Akt signaling as the most biologically coherent and clinically relevant pathways. HIF-1α plays a central role in renal adaptation to hypoxic stress, coordinating glycolytic reprogramming, mitophagy, and cell survival during ischemia–reperfusion. Experimental evidence demonstrates that HIF-1α is induced during reperfusion and is critical for proximal tubular cell survival, whereas its disruption exacerbates renal injury [46]. In parallel, PI3K–Akt signaling integrates survival and inflammatory cues in injured tubular cells and has been shown to attenuate ischemia–reperfusion injury via anti-apoptotic mechanisms [47]. Notably, prior studies report that AM mitigates cisplatin-induced renal cytotoxicity through modulation of PI3K/Akt signaling [47], supporting the biological plausibility of this pathway in our network. These findings suggest a coordinated HIF-1/PI3K–Akt axis that governs hypoxia adaptation and tubular survival. Enrichment for chemokine and infection-related pathways likely reflects shared inflammatory modules in infection and sterile renal injury rather than a direct antiviral role [48]. GO biological process enrichments (responses to chemicals/oxygenated compounds/organonitrogen, stress, intracellular signal transduction, and apoptosis) and molecular function (kinase activities, ATP/nucleotide binding, and oxidoreductases) closely mirror the KEGG results and are typical of datasets dominated by cytokine, kinase, and metabolic regulators. These patterns are consistent with canonical AKI biology: ischemia–reperfusion induces oxidative stress, inflammatory cytokine release, apoptotic signaling, and metabolic reprogramming in injured renal tubules, with chemokines, cytokines, and inflammasome-driven responses acting as core drivers of sterile renal injury [49]. GO cellular component terms clustered in vesicle and membrane microdomains showed an unusually strong signal for the γ-secretase complex genes (PSEN1/PSEN2/APH1A/APH1B/NCSTN) [50]. This finding is noteworthy because Notch activation, which requires γ-secretase cleavage, has been linked to both experimental AKI and kidney repair/fibrosis, and γ-secretase inhibitors can ameliorate injury and modulate inflammatory and renin–angiotensin pathways in murine AKI models [51]. The vesicles and exosomes signals align with growing evidence that extracellular vesicle contributes to ischemia/reperfusion (I/R)-induced tubular injury and intercellular communication in the injured kidney [52]. Network pharmacology is a valuable hypothesis-generating tool but has limitations. Target and pathway prioritization depend on the quality and coverage of interaction databases, and PPI networks may include missing, indirect or spurious links that affect hub detection and enrichment. Centrality metrics capture static topology and do not reflect dynamic, context-dependent biology, while results are sensitive to confidence thresholds and algorithmic choices. Therefore, prioritized targets and pathways require empirical validation (binding assays, cellular studies, and in vivo models) and should be interpreted as predictive rather than definitive.

Cyclooxygenase-2 (COX-2, encoded by *PTGS2*) is strongly implicated in the pathogenesis of acute kidney injury (AKI) [53]. COX-2 expression is markedly upregulated in renal tubular epithelial cells and infiltrating immune cells during injury, driving prostaglandin E2 (PGE2) production that modulates renal hemodynamics and inflammation [54]. While COX-2-derived prostaglandins can be protective under stress, excessive or dysregulated activity promotes tubular damage, inflammation, and progression from AKI to chronic kidney disease (CKD) [55]. Non-steroidal anti-inflammatory drugs (NSAIDs) and selective COX-2 inhibitors exert their effects by binding to COX-2 and suppressing prostaglandin synthesis. In the kidney, however, prostaglandins are essential for maintaining renal blood flow under stress conditions, particularly PGE2. Consequently, direct COX-2 inhibition can precipitate AKI, especially in vulnerable patients with dehydration, heart failure, or pre-existing CKD [56]. Celecoxib, a widely studied COX-2 inhibitor, can reduce inflammatory cytokines in renal models but also carries a risk of impairing renal perfusion [57]. Several natural compounds with COX-2 inhibitory activity are found in various medicinal plants, such as curcumin (turmeric), resveratrol (red grapes), quercetin (onions and apples), and polyphenols in green tea [58]. Some reviews suggest that natural COX-2 inhibitors may represent safer alternatives; however, further clinical studies are required to confirm their long-term safety [59]. Clinically, caution is advised in patients with CKD, diabetes mellitus, hypertension, or older age, and renal function should be monitored when these agents are combined with diuretics or renin–angiotensin–aldosterone system (RAAS) blockers [60]. The risk of AKI from natural compounds is generally lower than that from non-steroidal anti-inflammatory drugs (NSAIDs), as their inhibitory effects result in weaker COX-2 inhibition and act on multiple biological targets [61]. Previous studies showed that AM binds COX-2 within a pocket formed by residues ASN382, THR212, HIS207, ALA202, LEU298, VAL291, HIS386, HIS388, HIS214, and TYR385. The ligand forms four hydrogen bonds: two with ASN382 (ring A hydroxyls), one with THR212 (ring C hydroxyl), and one with HIS207 (ring B keto group), plus stabilizing π–π and π–alkyl hydrophobic interactions [62]. In contrast, our analysis revealed that AM was stably positioned within the catalytic cleft of COX-2, forming hydrogen bonds with HIS90, GLN192, SER353, PHE518, and SER530, along with extensive hydrophobic interactions involving VAL116, LEU352, LEU359, and LEU531 (Figure 5B). We also compared AM to rofecoxib, a selective COX-2 inhibitor, and found that rofecoxib occupied the same binding pocket in the COX-2 crystal structure [63]. AM and rofecoxib both anchor within the COX-2 active site by engaging key catalytic and hydrophobic residues. Their overlap at TYR385 and SER530 highlights a shared hydrogen bonding mechanism, while common hydrophobic contacts (VAL523, LEU531, and ALA527) suggest that AM exploits similar stabilizing interactions as rofecoxib (Figure 5B,C). TYR385 interacts with both ligands and plays a critical role in COX-2’s catalytic activity. These similarities support the notion that AM might act as a natural COX-2 inhibitor with a binding profile reminiscent of synthetic drugs. Natural COX-2 inhibitors may pose a lower risk of AKI than synthetic NSAIDs, with risk heightened in individuals with comorbidities or concurrent nephrotoxic drug use; dietary intake is usually safe, whereas supplemental use should be carefully monitored. MD simulation interaction analysis suggested that AM might establish stable contacts with key PTGS2 residues. Hydrogen bonds and water-bridge interactions were dominant with SER353, TYR355, ARG513, and SER530, while hydrophobic contacts were consistently observed with VAL523 and LEU531 (Figure 7C). Time-dependent analysis confirmed persistent interactions throughout the 200 ns trajectory, particularly with SER353, TYR355, and ARG513 (Figure 7D). The 2D interaction diagram further highlighted frequent hydrogen-bonding, hydrophobic, and polar contacts, with TYR355, ARG513, SER353, and SER530 showing the highest interaction frequencies (Figure 7E).

Tumor necrosis factor-α (TNF-α) is a central pro-inflammatory cytokine that is rapidly upregulated during AKI, promoting tubular epithelial cell apoptosis, leukocyte infiltration, and inflammatory signaling. Elevated circulating levels of its receptors, TNF receptor 1 (*TNFR1*) and TNF receptor 1 (*TNFR2*), have been associated with sustained kidney damage and progression toward fibrosis [64]. By contrast, AKT serine/threonine kinase 1 (*AKT1*), a key kinase in the PI3K/AKT pathway, plays a context-dependent role in AKI: transient AKT1 activation promotes tubular cell survival and repair, whereas prolonged or dysregulated activation can drive tubular dedifferentiation and fibrosis, promoting the progression from AKI to CKD [65]. TNF-mediated inflammation and AKT1-mediated signaling are interconnected contributors to AKI pathogenesis and its long-term sequelae, making key molecular targets for therapeutic intervention. Our docking results show that AM bound with favorable affinity to TNF and AKT1, suggesting it could concurrently modulate inflammatory signaling and cell-survival pathways, two central mechanisms in acute kidney injury. In TNF, the AM compound occupied the active site pocket, forming hydrogen bonds with GLN61 and hydrophobic interactions with residues including LEU57, TYR59, TYR19, TYR151, VAL123, and ILE155, thereby stabilizing the complex (Figure 6B). Previous studies reported that AM ameliorates glycerol-induced AKI by reducing elevated serum creatinine, BUN, magnesium, TNF-α, IL-6, renal edema, and lipid peroxidation, and improving renal histology, consistent with antioxidant and anti-inflammatory effects [9]. Anti-TNF biologics such as infliximab, etanercept, and adalimumab block TNF-α signaling and, in animal models of ischemia–reperfusion injury, reduce tubular apoptosis, suppress inflammation, and improve renal function [66]. However, they are not used clinically for AKI prevention due to infection risks and lack of trial evidence, while experimental small molecules like curcumin and resveratrol have shown indirect TNF suppression and renoprotective effects in AKI models [67,68]. Similarly, AM also docked into the AKT1 active site, forming hydrogen bonds with GLN79 and SER205 and hydrophobic contacts with TRP80, LEU210, LEU264, LYS268, VAL270, TYR272, and ILE290 (Figure 6D). Regarding AKT1, transient activation of the PI3K/AKT pathway supports tubular cell survival and repair, with compounds such as insulin-like growth factor-1 (IGF-1) and erythropoietin (EPO) demonstrating protective effects in preclinical AKI studies. In contrast, sustained AKT1 activation promotes fibrosis and tubular dedifferentiation, driving the progression from AKI to CKD [65]. AKT inhibitors have emerged as promising candidates for the treatment of AKI by targeting the PI3K/Akt pathway, a key regulator of inflammation, apoptosis, and fibrosis. Experimental studies demonstrate that AKT inhibition can attenuate inflammation through modulation of macrophage polarization, protect tubular cells from apoptosis, and slow the progression of AKI to CKD. Approaches under investigation include small molecules such as trametinib and triciribine, as well as genetic and RNA-based strategies to silence pathway components [69]. Therefore, AM demonstrates strong binding to both TNF and AKT1, key drivers of inflammation and fibrosis in acute kidney injury, suggesting its therapeutic potential to modulate interconnected signaling pathways and protect against AKI progression toward CKD.

The nephroprotective potential of AM is increasingly supported by a broad range of preclinical investigations. A recent systematic review and meta-analysis synthesizing in vivo and in vitro evidence confirmed that AM significantly reduces serum creatinine, blood urea nitrogen, malondialdehyde (MDA), reactive oxygen species (ROS), and pro-inflammatory cytokines, while improving antioxidant enzyme activity and renal histopathology in AKI models [9,11,12,70,71]. These findings are corroborated by glycerol-induced rhabdomyolysis models, where AM administration effectively lowered circulating TNF-α and IL-6, ameliorated renal edema, reduced lipid peroxidation, and restored tubular morphology [9]. Additional mechanistic insight is provided by cisplatin-induced nephrotoxicity studies in HEK293 cells, in which AM suppressed ROS overproduction, restored PI3K/Akt signaling, downregulated JNK activation, and inhibited caspase-mediated apoptosis—demonstrating a clear molecular basis for its antioxidative and cytoprotective actions [12]. Beyond AKI, AM has also demonstrated renoprotective effects in metabolic disease models such as type II diabetes mellitus, where treatment improved creatinine levels and normalized structural kidney damage, suggesting broader applicability to renal pathology beyond acute injury [72]. Furthermore, its anti-inflammatory properties are repeatedly substantiated in macrophage models through significant suppression of TNF-α, IL-1β, and IL-6 production, supporting AM’s strong immunomodulatory profile [62]. Taken together, these expanded findings reinforce the coherence between our network pharmacology predictions—particularly the centrality of TNF, IL6, AKT1, PTGS2, HSP90AA1, and NFKB1—and the experimentally validated antioxidant, anti-inflammatory, and cytoprotective effects of AM. This convergence of computational and empirical evidence strongly strengthens the plausibility of AM as a multi-target therapeutic candidate for AKI and other renal disorders.

Previous in vivo and in vitro studies have reported AM’s antioxidative and anti-inflammatory effects in AKI models [9,11,12]. However, those reports focus on individual pathways or models and do not systematically map AM’s multi-target interactions in the context of AKI. Our study fills this gap by integrating three complementary target-prediction platforms with disease–gene collections, PPI topology, pathway enrichment and structural docking to: (1) generate an ordered, testable list of AM–AKI candidate targets (122 intersecting genes) and prioritize ten consensus hub genes (*TNF*, *AKT1*, *IL6*, *SRC*, *CTNNB1*, *HSP90AA1*, *NFKB1*, *HIF1A*, *PPARG*, and *PTGS2*) supported across data layers; (2) highlight pathway clusters (HIF-1, PI3K–AKT, chemokine signaling, AGE–RAGE, and cellular senescence) that unify disparate mechanistic observations from earlier studies; (3) provide molecular-level hypotheses by demonstrating favorable docking of AM to key hub proteins (notably PTGS2, TNF, and AKT1) and identifying plausible binding residues for experimental validation. This integrated view helps to prioritize biologically meaningful targets and pathways for future experimental work, thereby refining and extending current understanding of AM’s potential roles in AKI.

Future research on AM should address several critical areas to enhance its therapeutic potential in AKI. First, in vivo studies are essential to validate the efficacy and safety of AM in clinically relevant models of AKI, assessing its effects on renal function, histology, and biochemical markers of injury. Second, investigations into advanced formulation techniques, such as nanoparticle encapsulation or lipid-based delivery systems, are necessary to improve the bioavailability and solubility of AM, enabling more effective therapeutic concentrations. Third, mechanistic studies using transcriptomics and proteomics can clarify pathways modulated by AM and identify pharmacodynamic biomarkers for patient stratification. Finally, expanding the research to include combinations of AM with other agents, such as existing anti-inflammatory or nephroprotective drugs, might yield synergistic effects, enhancing treatment outcomes for AKI.

## 5. Conclusions

This study demonstrates that AM holds significant potential as a multi-target therapeutic agent for AKI. Through a comprehensive approach combining network pharmacology and molecular docking, we identified key protein interactions and signaling pathways associated with AM, particularly its effects on inflammation and cell-survival mechanisms. The findings suggest that AM can modulate critical pathways such as PI3K-AKT and HIF-1 signaling, which are pivotal in the renal response to ischemia and oxidative stress. Additionally, the strong binding affinity of AM for *PTGS2* reinforces its role in mitigating inflammation. However, these findings are predictive: network analyses, enrichment results and docking depend on database quality, algorithmic assumptions and structural approximations and cannot capture full biological complexity. Therefore, the results should be considered hypothesis-generating and require experimental validation before translational conclusions.

## Figures and Tables

**Table 1:** Details of the protein targets in the PDB database and the grid docking parameters in molecular docking simulation.

Targets	PDB ID	Method	Co-Ligand/Drug	Resolution (Å)	R-Value Free	R-Value Work	Spacing (Å)	Grid Box Size (in XYZ)	Center Grid Box		
									X Center	Y Center	Z Center
*AKT1*	3O96	X-ray diffraction	IQO0444	2.70 Å	0.308	0.245	0.375	90 × 90 × 100	6.29	−7.942	16.262
*IL6*	1ALU	X-ray diffraction	BMS1166	1.90 Å	0.277	0.213	0.375	100 × 114 × 100	1.251	−19.933	8.838
*SRC*	2BDJ	X-ray diffraction	HET800	2.50 Å	0.277	0.158	0.375	70 × 70 × 70	15.578	0.187	25.295
*CTNNB1*	4DJS	X-ray diffraction	Tegatrabetan	3.03 Å	0.291	0.263	0.903	60 × 60 × 126	−1.641	8.22	−38.836
*HSP90AA1*	7UR3	X-ray diffraction	OJ3301	1.60 Å	0.189	0.174	0.375	80 × 80 × 80	−1.455	−11.372	−4.151
*NFKB1*	8TQD	X-ray diffraction	JMR301	2.02 Å	0.227	0.182	0.375	100 × 80 × 80	6.419	−4.072	−4.922
*HIF1A*	8II0	X-ray diffraction	P5I1001	2.04 Å	0.239	0.205	0.375	70 × 70 × 70	21.412	−28.028	2.769
*PPARG*	7QB1	X-ray diffraction	9WQ501	2.20 Å	0.249	0.199	0.375	70 × 70 × 80	16.77	18.821	8.217
*PTGS2*	5KIR (Human)	X-ray diffraction	Rofecoxib	2.70 Å	0.220	0.178	0.375	70 × 70 × 70	24.984	3.735	34.517
	3LN1 (*Mus musculus*)	X-ray diffraction	Celecoxib	2.40 Å	0.264	0.232	0.375	60 × 60 × 60	32.298	−23.859	−15.352
*TNF*	7KPB	X-ray diffraction	D84201	3.00 Å	0.256	0.223	0.375	80 × 80 × 80	−58.842	91.595	−6.671

*AKT1*: AKT serine/threonine kinase 1; *IL6*: interleukin-6; *SRC*: SRC proto-oncogene, non-receptor tyrosine kinase; *CTNNB1*: catenin beta-1; *HSP90AA1*: heat shock protein 90 alpha family class A member 1; *NFKB1*: nuclear factor kappa B subunit 1; *HIF1A*: hypoxia-inducible factor 1-alpha; *PPARG*: peroxisome proliferator-activated receptor gamma; *PTGS2*: prostaglandin-endoperoxide synthase 2 (COX-2); *TNF*: tumor necrosis factor; PDB: Protein Data Bank; resolution (Å): structure resolution in angstroms; spacing (Å): grid spacing for docking; grid box size (XYZ): docking grid dimensions; center grid box (X, Y, Z): coordinates of grid box center; co-ligand/drug: ligand present in the deposited PDB structure.

**Table 2:** The 122 overlapping targets between alpha-mangostin (AM) and acute kidney injury (AKI).

No.	Common Name	Target
1	*ABCB1*	ATP binding cassette subfamily B member 11
2	*ABCG2*	ATP binding cassette subfamily G member 2
3	*ABL1*	ABL proto-oncogene 1, non-receptor tyrosine kinase
4	*ACHE*	Acetylcholinesterase
5	*ACP1*	Acid phosphatase 1
6	*ADCYAP1R1*	ADCYAP receptor type I
7	*AGTR1*	Angiotensin II receptor type 1
8	*AKT1*	AKT serine/threonine kinase 1
9	*ALOX12*	Arachidonate 12-lipoxygenase
10	*ALOX15*	Arachidonate 15-lipoxygenase
11	*ALOX5*	Arachidonate 5-lipoxygenase
12	*APH1A*	Aph-1 homolog A, gamma-secretase subunit
13	*APH1B*	Aph-1 homolog B, gamma-secretase subunit
14	*APP*	Amyloid beta precursor protein
15	*AXL*	AXL receptor tyrosine kinase
16	*BCHE*	Butyrylcholinesterase
17	*BCL2L1*	BCL2 like 1
18	*CALCA*	Calcitonin-related polypeptide alpha
19	*CASP1*	Caspase 1
20	*CBR1*	Carbonyl reductase 1
21	*CCND1*	Cyclin D1
22	*CCR1*	C-C motif chemokine receptor 1
23	*CCR4*	C-C motif chemokine receptor 4
24	*CCR5*	C-C motif chemokine receptor 5
25	*CDK2*	Cyclin dependent kinase 2
26	*CDK4*	Cyclin dependent kinase 4
27	*CFTR*	Cystic fibrosis transmembrane conductance regulator
28	*CHEK1*	Checkpoint kinase 1
29	*CHEK2*	Checkpoint kinase 2
30	*CHUK*	Component of inhibitor of nuclear factor kappa B kinase complex
31	*COMT*	Catechol-O-methyltransferase
32	*CREB1*	CAMP responsive element binding protein 1
33	*CTNNB1*	Catenin beta 1
34	*CTSV*	Cathepsin V
35	*CXCR1*	C-X-C motif chemokine receptor 1
36	*CXCR2*	C-X-C motif chemokine receptor 2
37	*CYP19A1*	Cytochrome P450 family 19 subfamily A member 1
38	*CYP1A1*	Cytochrome P450 family 1 subfamily A member 1
39	*CYP1B1*	Cytochrome P450 family 1 subfamily B member 1
40	*DHFR*	Dihydrofolate reductase
41	*DRD2*	Dopamine receptor D2
42	*DRD3*	Dopamine receptor D3
43	*EGLN1*	Egl-9 family hypoxia-inducible factor 1
44	*ELAVL3*	ELAV-like RNA-binding protein 3
45	*ERBB2*	Erb-B2 receptor tyrosine kinase 2
46	*F10*	Coagulation factor X
47	*F2*	Coagulation factor II
48	*FASN*	Fatty acid synthase
49	*GCGR*	Glucagon receptor
50	*GLO1*	Glyoxalase I
51	*GSK3B*	Glycogen synthase kinase 3 beta
52	*HDAC1*	Histone deacetylase 1
53	*HIF1A*	Hypoxia-inducible factor 1 subunit alpha
54	*HSD17B1*	Hydroxysteroid 17-beta dehydrogenase 1
55	*HSP90AA1*	Heat shock protein 90 alpha family class A member 1
56	*HSP90AB1*	Heat shock protein 90 alpha family class B member 1
57	*HSP90B1*	Heat shock protein 90 beta family member 1
58	*IDH1*	Isocitrate dehydrogenase (NADP(+)) 1
59	*IKBKB*	Inhibitor of nuclear factor kappa B kinase subunit beta
60	*IL6*	interleukin 6
61	*IMPDH1*	Inosine monophosphate dehydrogenase 1
62	*IMPDH2*	Inosine monophosphate dehydrogenase 2
63	*ITGA4*	Integrin subunit alpha 4
64	*ITGB1*	Integrin subunit beta 1
65	*KCNH2*	Potassium voltage-gated channel subfamily H member 2
66	*KDM1A*	Lysine demethylase 1A
67	*KISS1R*	KISS1 receptor
68	*KLK1*	Kallikrein-related peptidase 1
69	*KLK2*	Kallikrein-related peptidase 2
70	*KLKB1*	Kallikrein B1
71	*LDHA*	Lactate dehydrogenase A
72	*LDHB*	Lactate dehydrogenase B
73	*MAOA*	Monoamine oxidase A
74	*MAP2K1*	Mitogen-activated protein kinase kinase 1
75	*MAPK1*	Mitogen-activated protein kinase 1
76	*MAPK3*	Mitogen-activated protein kinase 3
77	*MPG*	N-methylpurine DNA glycosylase
78	*MTOR*	Mechanistic target of rapamycin kinase
79	*NCSTN*	Nicastrin
80	*NFKB1*	Nuclear factor kappa B subunit 1
81	*NQO1*	NAD(P)H quinone dehydrogenase 1
82	*ODC1*	Ornithine decarboxylase 1
83	*OPRK1*	Opioid receptor kappa 1
84	*PCSK7*	Proprotein convertase subtilisin/kexin type 7
85	*PDE4D*	Phosphodiesterase 4D
86	*PDK1*	Pyruvate dehydrogenase kinase 1
87	*PIK3CA*	Phosphatidylinositol-4,5-bisphosphate 3-kinase catalytic subunit alpha
88	*PLAU*	Plasminogen activator, urokinase
89	*PLK1*	Polo like kinase 1
90	*PPARG*	Peroxisome proliferator-activated receptor gamma
91	*PRKCA*	Protein kinase C alpha
92	*PRKCB*	Protein kinase C beta
93	*PRKCD*	Protein kinase C delta
94	*PRKCE*	Protein kinase C epsilon
95	*PRKCG*	Protein kinase C gamma
96	*PRKCH*	Protein kinase C eta
97	*PRKCI*	Protein kinase C iota
98	*PRSS1*	Serine protease 1
99	*PSEN1*	Presenilin 1
100	*PSEN2*	Presenilin 2
101	*PSENEN*	Presenilin enhancer, gamma-secretase subunit
102	*PTGES*	Prostaglandin E synthase
103	*PTGS1*	Prostaglandin-endoperoxide synthase 1
104	*PTGS2*	Prostaglandin-endoperoxide synthase 2
105	*PTPN1*	Protein tyrosine phosphatase non-receptor type 1
106	*PTPRS*	Protein tyrosine phosphatase receptor type S
107	*RARA*	Retinoic acid receptor alpha
108	*RELA*	RELA proto-oncogene, NF-KB subunit
109	*RPS6KA3*	Ribosomal protein S6 kinase A3
110	*SERPINE1*	Serpin family E member 1
111	*SIRT1*	Sirtuin 1
112	*SQLE*	Squalene epoxidase
113	*SRC*	SRC proto-oncogene, non-receptor tyrosine kinase
114	*STAT6*	Signal transducer and activator of transcription 6
115	*TCF4*	Transcription factor 4
116	*THRB*	Thyroid hormone receptor beta
117	*TNF*	Tumor necrosis factor
118	*TNNC1*	Troponin C1, slow skeletal and cardiac type
119	*TNNI3*	Troponin I3, cardiac type
120	*TNNT2*	Troponin T2, cardiac type
121	*TYR*	Tyrosinase
122	*VCP*	Valosin-containing protein

**Table 3:** Kyoto Encyclopedia of Genes and Genomes (KEGG) pathway enrichment analysis of 122 overlapping genes associated with alpha-mangostin (AM) and acute kidney injury (AKI).

Pathway	Number of Genes	Pathway Genes	Fold Enrichment	Enrichment FDR
hsa05200 pathways in cancer	36	530	12.77	1.8235 × 10^−27^
hsa05215 prostate cancer	20	97	38.67	1.6478 × 10^−24^
hsa05163 human cytomegalovirus infection	24	224	20.09	1.0457 × 10^−22^
hsa05167 Kaposi sarcoma-associated herpesvirus infection	22	194	21.27	2.2257 × 10^−21^
hsa04066 HIF-1 signaling pathway	18	109	30.97	1.8455 × 10^−20^
hsa05206 microRNAs in cancer	20	161	23.30	2.5219 × 10^−20^
hsa05207 chemical carcinogenesis—receptor activation	20	197	19.04	1.3238 × 10^−18^
hsa04151 PI3K-Akt signaling pathway	24	354	12.72	2.1729 × 10^−18^
hsa01521 EGFR tyrosine kinase inhibitor resistance	15	79	35.61	3.7204 × 10^−18^
hsa04919 thyroid hormone signaling pathway	16	121	24.80	7.5875 × 10^−17^
hsa05212 pancreatic cancer	14	76	34.55	7.5875 × 10^−17^
hsa05220 chronic myeloid leukemia	14	76	34.55	7.5875 × 10^−17^
hsa05165 human papillomavirus infection	22	331	12.47	7.8795 × 10^−17^
hsa05417 lipid and atherosclerosis	19	214	16.65	7.8795 × 10^−17^
hsa04933 AGE-RAGE signaling pathway in diabetic complications	15	100	28.13	9.5621 × 10^−17^
hsa05208 chemical carcinogenesis—reactive oxygen species	19	223	15.98	1.5011 × 10^−16^
hsa04062 chemokine signaling pathway	18	191	17.67	1.7834 × 10^−16^
hsa05161 hepatitis B	17	162	19.68	2.2658 × 10^−16^
hsa05135 Yersinia infection	16	137	21.90	3.5317 × 10^−16^
hsa04218 cellular senescence	16	156	19.24	2.7661 × 10^−15^

hsa: *Homo sapiens* KEGG pathway code; pathway genes: total number of genes annotated to the pathway; fold enrichment: ratio of observed gene frequency to expected background frequency; FDR: false discovery rate. Pathway names: HIF-1 = hypoxia-inducible factor-1; PI3K-Akt = phosphoinositide-3-kinase/protein kinase B signaling pathway signaling; EGFR = epidermal growth factor receptor; AGE-RAGE = advanced glycation end products and receptor of advanced glycation end products.

**Table 4:** GO biological process enrichment analysis of 122 overlapping genes associated with alpha-mangostin (AM) and acute kidney injury (AKI).

Pathway	Number of Genes	Pathway Genes	Fold Enrichment	Enrichment FDR
GO:0042221 response to chemical	93	4821	3.62	3.9104 × 10^−35^
GO:0070887 cellular response to chemical stimulus	80	3300	4.55	1.0511 × 10^−34^
GO:1901700 response to oxygen-containing compound	63	1752	6.74	1.2268 × 10^−34^
GO:0010033 response to organic substance	78	3269	4.48	3.3062 × 10^−33^
GO:1901698 response to nitrogen compound	53	1172	8.48	5.8736 × 10^−33^
GO:0010243 response to organonitrogen compound	50	1061	8.84	1.2324 × 10^−31^
GO:0065008 reg. of biological quality	83	4103	3.79	2.5301 × 10^−31^
GO:1901701 cellular response to oxygen-containing compound	53	1272	7.81	2.5301 × 10^−21^
GO:0071310 cellular response to organic substance	65	2609	4.67	5.4778 × 10^−27^
GO:0014070 response to organic cyclic compound	44	958	8.61	6.5434 × 10^−27^
GO:0006950 response to stress	79	4424	3.35	2.5691 × 10^−25^
GO:0009719 response to endogenous stimulus	52	1660	5.88	7.7148 × 10^−25^
GO:1901699 cellular response to nitrogen compound	38	734	9.71	1.1898 × 10^−24^
GO:0051239 regulation of multicellular organismal process	66	3024	4.09	2.3329 × 10^−24^
GO:0071417 cellular response to organonitrogen compound	36	654	10.32	3.7985 × 10^−24^
GO:0035556 intracellular signal transduction	64	2847	4.22	4.0074 × 10^−24^
GO:0008219 cell death	58	2320	4.69	1.6412 × 10^−23^
GO:0032879 regulation of localization	64	2945	4.08	2.3041 × 10^−23^
GO:0044093 positive reg. of molecular function	51	1718	3.62	2.3041 × 10^−23^
GO:0006915 apoptotic process	55	2065	4.55	2.3181 × 10^−23^

GO: Gene Ontology; FDR: false discovery rate.

**Table 5:** GO molecular function enrichment analysis of 122 overlapping genes associated with alpha-mangostin (AM) and acute kidney injury (AKI).

Pathway	Number of Genes	Pathway Genes	Fold Enrichment	Enrichment FDR
GO:0042221 response to chemical	54	2237	4.5273	2.3224 × 10^−20^
GO:0070887 cellular response to chemical stimulus	28	632	8.3091	8.1332 × 10^−16^
GO:1901700 response to oxygen-containing compound	25	470	9.9760	8.1332 × 10^−16^
GO:0010033 response to organic substance	51	2743	3.4871	1.3988 × 10^−14^
GO:1901698 response to nitrogen compound	28	748	7.0206	3.3639 × 10^−14^
GO:0010243 response to organonitrogen compound	21	376	10.4748	9.5501 × 10^−14^
GO:0065008 reg. of biological quality	48	2577	3.4933	9.5501 × 10^−14^
GO:1901701 cellular response to oxygen-containing compound	45	2381	3.5446	4.3208 × 10^−13^
GO:0071310 cellular response to organic substance	22	471	8.7603	4.3208 × 10^−13^
GO:0014070 response to organic cyclic compound	28	849	6.1854	4.3208 × 10^−13^
GO:0006950 response to stress	45	2382	3.5431	4.3208 × 10^−13^
GO:0009719 response to endogenous stimulus	47	2630	3.3516	6.2357 × 10^−13^
GO:1901699 cellular response to nitrogen compound	28	1008	5.2097	2.1554 × 10^−14^
GO:0051239 regulation of multicellular organismal process	42	2342	3.3634	2.2744 × 10^−11^
GO:0071417 cellular response to organonitrogen compound	25	820	5.7180	5.5003 × 10^−11^
GO:0035556 intracellular signal transduction	35	1729	3.7965	1.1345 × 10^−10^
GO:0008219 cell death	7	18	72.9358	1.2242 × 10^−10^
GO:0032879 regulation of localization	35	1741	3.7704	1.2278 × 10^−10^
GO:0044093 positive regulation of molecular function	34	1662	3.8367	1.6054 × 10^−10^
GO:0006915 apoptotic process	24	824	5.4626	3.0854 × 10^−10^

GO: Gene Ontology, FDR: false discovery rate.

**Table 6:** GO cellular component enrichment analysis of 122 overlapping genes associated with alpha-mangostin (AM) and acute kidney injury (AKI).

Pathway	Number of Genes	Pathway Genes	Fold Enrichment	Enrichment FDR
GO:0031982 vesicle	63	4466	2.65	9.3242 × 10^−13^
GO:0070765 gamma-secretase complex	6	7	160.76	3.2603 × 10^−11^
GO:0048471 perinuclear region of cytoplasm	25	770	6.09	4.7487 × 10^−11^
GO:0031410 cytoplasmic vesicle	45	2849	2.96	4.0581 × 10^−10^
GO:0097708 intracellular vesicle	45	2851	2.96	4.0581 × 10^−10^
GO:0030054 cell junction	39	2293	3.19	1.7483 × 10^−9^
GO:0150034 distal axon	14	286	9.18	2.7869 × 10^−8^
GO:0045121 membrane raft	15	351	8.01	3.3847 × 10^−8^
GO:0098857 membrane microdomain	15	352	7.99	3.3847 × 10^−8^
GO:0009986 cell surface	24	1050	4.29	6.1861 × 10^−8^
GO:0036477 somatodendritic compartment	22	888	4.65	6.3891 × 10^−8^
GO:0045202 synapse	28	1435	3.66	6.3891 × 10^−8^
GO:0099503 secretory vesicle	25	1165	4.02	7.3919 × 10^−8^
GO:0005739 mitochondrion	31	1830	3.18	1.6923 × 10^−7^
GO:0098590 plasma membrane region	26	1332	3.66	2.0723 × 10^−7^
GO:0099056 integral component of presynaptic membrane	8	76	19.74	2.0788 × 10^−7^
GO:0005615 extracellular space	45	3577	2.36	2.0942 × 10^−7^
GO:0070062 extracellular exosome	35	2316	2.83	2.0942 × 10^−7^
GO:0043230 extracellular organelle	35	2343	2.80	2.3066 × 10^−7^
GO:0065010 extracellular membrane-bounded organelle	35	2343	2.80	2.3066 × 10^−7^

GO: Gene Ontology, FDR: false discovery rate.

**Table 7:** Computational docking evaluation of alpha-mangostin (AM) against molecular targets in acute kidney injury (AKI).

No	Protein Name	PDB	Compound and Positive Control	Binding Energies (kcal/mol)	Inhibition Constant (nM)	RMSD (Å)
1	AKT1	3O96	Alpha-mangostin	−9.48	112.82	-
			IQO0444 (Co-crystallized)	−12.57	0.60799	1.07
2	IL6	1ALU	Alpha-mangostin	−6.67	12,920.00	-
			BMS1166 (PubChemCID_118434635)	−7.61	2620.00	-
3	SRC	2BDJ	Alpha-mangostin	−8.64	464.16	-
			HET800 (Co-crystallized)	−10.25	29.66	0.89
4	CTNNB1	4DJS	Alpha-mangostin	−4.76	32,188.00	-
			Tegatrabetan (BC-2059) (PubChemCID_91193182)	−7.51	3130.00	-
5	HSP90AA1	7UR3	Alpha-mangostin	−9.16	193.81	-
			OJ3301 (Co-crystallized)	−13.31	0.17422	0.75
6	NFKB1	8TQD	Alpha-mangostin	−7.31	4350.00	-
			JMR301 (Co-crystallized)	−6.61	14,280.00	3.00
7	HIF1A	8II0	Alpha-mangostin	−8.88	309.33	-
			P5I1001 (Co-crystallized)	−12.24	1.06	2.64
8	PPARG	7QB1	Alpha-mangostin	−8.24	909.09	-
			9WQ501 (Co-crystallized)	−9.76	70.24	1.72
9	PTGS2	5KIR (Human)	Alpha-mangostin	−11.13	6.95	-
			Rofecoxib (Co-crystallized)	−10.55	18.63	1.40
		3LN1 (*Mus musculus*)	Alpha-mangostin	−10.94	9.64	-
			Celecoxib (Co-crystallized)	−10.84	11.32	0.95
10	TNF	7KPB	Alpha-mangostin	−9.74	72.51	-
			D84201 (Co-crystallized)	−13.67	0.0915	1.46

PDB: Protein Data Bank; binding energy: predicted binding affinity between ligand and protein (kcal/mol); inhibition constant (Ki): estimated inhibition constant calculated from binding energy (nM). Protein names: AKT1, AKT serine/threonine kinase 1; IL6, interleukin-6; SRC, SRC proto-oncogene, non-receptor tyrosine kinase; CTNNB1, catenin beta-1; HSP90AA1, heat shock protein 90 alpha family class A member 1; NFKB1, nuclear factor kappa B subunit 1; HIF1A, hypoxia-inducible factor 1-alpha; PPARG, peroxisome proliferator-activated receptor gamma; PTGS2, prostaglandin-endoperoxide synthase 2 (COX-2); TNF, tumor necrosis factor. Positive controls: IQO0444 (AKT1 inhibitor), BMS1166 (IL-6 inhibitor), HET800 (SRC ligand), Tegatrabetan/BC-2059 (CTNNB1 inhibitor), OJ3301 (HSP90 inhibitor), JMR301 (NFKB1 modulator), P5I1001 (HIF1A inhibitor), 9WQ501 (PPARG agonist), Rofecoxib (selective COX-2 inhibitor), D84201 (TNF inhibitor).

## Data Availability

The original contributions presented in this study are included in the article/Supplementary Materials. Further inquiries can be directed to the corresponding author.

## Supplementary Materials

The following supporting information can be downloaded at: https://www.mdpi.com/article/10.3390/foods15071270/s1, Table S1: Comparison of ADMET results: SwissADME vs. PkCSM.

## Abbreviations

The following abbreviations are used in this manuscript:

ADMET	Absorption, distribution, metabolism, excretion, and toxicity
ADT	AutoDockTools
AGE-RAGE	Advanced glycation end products and receptor of advanced glycation end products
AKI	Acute kidney injury
*AKT1*	AKT serine/threonine kinase 1 (protein kinase B)
AM	Alpha-mangostin
AMES	Ames bacterial reverse mutation test (mutagenicity assay)
BBB	Blood–brain barrier
BUN	Blood urea nitrogen
CKD	Chronic kidney disease
COX-2	Cyclooxygenase-2 (enzyme; gene *PTGS2*)
CNS	Central nervous system
CYP450	Cytochrome P450
DC	Degree centrality
EGFR	Epidermal growth factor receptor
EPO	Erythropoietin
FDR	False discovery rate
GI	Gastrointestinal
GO	Gene Ontology
HBD	Hydrogen-bond donor
HBA	Hydrogen-bond acceptor
*HIF-1*	Hypoxia-inducible factor-1
*HSP90AA1*	Heat shock protein 90 alpha family class A member 1
*IL-6*	Interleukin-6
*IGF-1*	Insulin-like growth factor-1
*JNK*	c-Jun N-terminal kinase
KEGG	Kyoto Encyclopedia of Genes and Genomes
Ki	Inhibition constant
Log P	Partition coefficient (lipophilicity)
*MAPK*	Mitogen-activated protein kinase
*NFKB1*	Nuclear factor kappa B subunit 1
PAINS	Pan-assay interference substances
PDB	Protein Data Bank
PI3K	Phosphoinositide 3-kinase
PI3K-Akt	Phosphatidylinositol 3-kinase–protein kinase B signaling pathway
P-gp	P-glycoprotein
*PGE2*	Prostaglandin E2
*PTGS2*	Prostaglandin-endoperoxide synthase 2 (COX-2 gene)
RAASå	Renin–angiotensin–aldosterone system
ROS	Reactive oxygen species
SEA	Similarity Ensemble Approach
SMILES	Simplified molecular-input line-entry system
TNF	Tumor necrosis factor
TNF-α	Tumor necrosis factor-alpha
*TNFR1*	TNF receptor 1
*TNFR2*	TNF receptor 2
TPSA	Topological polar surface area
