# Supplementary material for: Integrative Network Pharmacology and Molecular Docking Analysis Uncovers Multi-Target Mechanisms of Alpha-Mangostin Against Acute Kidney Injury
Source: Foods. 2026 Apr 7;15(7):1270. doi: 10.3390/foods15071270 (PMC13073608; doi:10.3390/foods15071270)
Supplement: Supplementary file 1 [file foods-15-01270-s001.zip › foods-4214748-supplementary.pdf]

Integrative Network Pharmacology and Molecular Docking Analysis Uncovers Multi-Target Mechanisms of Alpha-Mangostin Against Acute Kidney Injury

Moragot Chatatikun [1,2], Aman Tedasen [1,2], Chutima Jansakun [1], Passakorn Poolbua [1], Jason C. Huang [3], Jongkonnee Thanasai [4], Wiyada Kwanhian Klangbud [5], Atthaphong Phongphithakchai [6,*]

[1] Department of Medical Technology, School of Allied Health Sciences, Walailak University, Nakhon Si Thammarat 80160, Thailand; moragot.ch@wu.ac.th (M.C.); aman.te@wu.ac.th (A.T.); chutima.js@wu.ac.th (C.J.); patsakorn@mail.wu.ac.th (P.P.)

[2] Research Excellence Center for Innovation and Health Products (RECIHP), Walailak University, Nakhon Si Thammarat 80160, Thailand

[3] Department of Biotechnology and Laboratory Science in Medicine, National Yang Ming Chiao Tung University, Taipei 112304, Taiwan; jasonhuang@nycu.edu.tw

[4] Faculty of Medicine, Mahasarakham University, Mahasarakham 44000, Thailand; jongkonnee@msu.ac.th

[5] Medical Technology Program, Faculty of Science, Nakhon Phanom University, Nakhon Phanom 48000, Thailand; wiyadakwanhian@gmail.com

[6] Nephrology Unit, Division of Internal Medicine, Faculty of Medicine, Prince of Songkla University, Songkhla 90110, Thailand

* Correspondence: atthaphong.p@psu.ac.th

Supplementary Table S1. Comparison of ADMET results: SwissADME vs PkCSM.

Property	SwissADME	pkCSM
**Physicochemical properties**		
Number of heavy atoms	30	N/A
Number of aromatic heavy atoms	14	N/A
Fraction Csp3	0.29	N/A
Number of H-bond acceptors	6	N/A
Number of H-bond donors	3	N/A
Number of rotatable bonds	5	N/A
TPSA	100.13 Å^2	N/A
**Lipophilicity**		
Log $P_{o/w}$ (iLOGP)	4.14	N/A
Log $P_{o/w}$ (XLOGP3)	6.27	N/A
Log $P_{o/w}$ (WLOGP)	5.09	N/A
Log $P_{o/w}$ (MLOGP)	2.19	N/A
Log $P_{o/w}$ (SILICOS-IT)	5.52	N/A
Log $P_{o/w}$ (iLOGP)	4.14	N/A
Consensus Log $P_{o/w}$	4.64	N/A
**Water Solubility**		
Log *S* (ESOL)	-6.35	
Solubility	1.83e-04 mg/mL ; 4.46e-07 mol/L	N/A
Class	Poorly soluble	N/A
Log S (Ali)	-8.16	N/A
Solubility	2.84e-06 mg/mL ; 6.91e-09 mol/L	N/A
Class	Poorly soluble	N/A
Log S (SILICOS-IT)	-6.14	N/A
Solubility	2.97e-04 mg/mL ; 7.23e-07 mol/L	N/A

Class	Poorly soluble	N/A
**Pharmacokinetics**		
**Asorption**		
Caco2 permeability (log Papp in 10^{-6} cm/s)	N/A	-0.048
GI absorption (% Absorbed)	High	93.647
Skin permeability	-4.35	-2.736
P-glycoprotein substrate	No	Yes
P-glycoprotein I inhibitor	N/A	Yes
P-glycoprotein II inhibitor	N/A	Yes
**Distribution**		
VDss (human) (log L/kg)	N/A	-0.282
Fraction unbound (human)	N/A	≈ 0
BBB permeant (log BB)	No	-1.075
CNS permeability (log PS)	N/A	-1.984
**Metabolism**		
CYP2D6 substrate	N/A	No
CYP3A4 substrate	N/A	Yes
CYP1A2 inhibitor	No	Yes
CYP2C19 inhibitor	No	Yes
CYP2C9 inhibitor	Yes	Yes
CYP2D6 inhibitor	No	No
CYP3A4 inhibitor	No	No
**Excretion**		
Total clearance	N/A	0.43
Renal OCT2 Substrate	N/A	No
**Toxicity**		
AMES toxicity	N/A	Yes

Max. tolerated dose (human) (log mg/kg/day)	N/A	0.061
hERG I inhibitor	N/A	No
hERG II inhibitor	N/A	Yes
Oral Rat Acute Toxicity (LD50) (mol/kg)	N/A	1.949
Oral Rat Chronic Toxicity (LOAEL) (log mg/kg_bw/day)	N/A	1.594
Hepatotoxicity	N/A	No
Skin Sensitisation	N/A	No
T. Pyriformis toxicity (log ug/L)	N/A	0.325
Minnow toxicity (log mM)	N/A	-0.138
**Druglikeness**		
Lipinski	Yes; 0 violation	N/A
Ghose	Yes	N/A
Veber	Yes	N/A
Egan	Yes	Egan
Muegge	No; 1 violation: XLOGP3>5	Muegge
Bioavailability Score	0.55	Bioavailability Score
**Medinical chemistry**		
PAINS	0 alert	N/A
Brenk	2 alerts: isolated_alkene, polycyclic_aromatic_hydrocarbon_2	N/A
Leadlikeness	No; 2 violations: MW>350, XLOGP3>3.5	N/A
Synthetic accessibility	3.91	N/A
